# Evaluation of a method for calculating carboplatin dosage in DeVIC ± R therapy (combination therapy of dexamethasone, etoposide, ifosfamide and carboplatin with or without rituximab) as a salvage therapy in patients with relapsed or refractory non-Hodgkin lymphoma

**DOI:** 10.1007/s00280-016-3076-9

**Published:** 2016-06-20

**Authors:** Ayana Tomono, Kaori Ito, Takahiro Hayashi, Maiko Ando, Yosuke Ando, Masahiro Tsuge, Akinao Okamoto, Yoko Inaguma, Masataka Okamoto, Nobuhiko Emi, Shigeki Yamada

**Affiliations:** 1 Department of Pharmacy, Fujita Health University Hospital, 1-98 Dengakugakubo, Kutsukake-cho, Toyoake, 470-1192 Japan; 2 Department of Hematology, School of Medicine, Fujita Health University, 1-98 Dengakugakubo, Kutsukake-cho, Toyoake, 470-1192 Japan; 3 Department of Clinical Pharmacy, School of Medicine, Fujita Health University, 1-98 Dengakugakubo, Kutsukake-cho, Toyoake, 470-1192 Japan

**Keywords:** DeVIC ± R therapy, Dose calculation method, CBDCA, Non-Hodgkin lymphoma, Calvert formula

## Abstract

**Purpose:**

Several studies have evaluated the utility of extrapolating the Calvert formula in calculating carboplatin (CBDCA) dosages in solid tumours; however, data regarding haematological cancers are less. Therefore, we conducted a preliminary study of the utility of extrapolating the Calvert formula in calculating CBDCA dosages for DeVIC ± R therapy.

**Methods:**

A retrospective study on 57 non-Hodgkin lymphoma patients who had received DeVIC ± R therapy was conducted. The area under the curve (AUC) of CBDCA was back-calculated from actual dosages using the Calvert formula. Patients were divided into two groups according to an AUC ≥ 4 or an AUC < 4, respectively. The Revised Response Criteria of the International Working Group and CTCAE version 4.0 were used for assessing the treatment efficacy and adverse events, respectively.

**Results:**

The use of AUC instead of body surface area had greater utility in calculating CBDCA dosage, with a response rate of greater than 50 % in patients receiving DeVIC ± R therapy with an AUC ≥ 4 for CBDCA. The response rate of the AUC ≥ 4 group was significantly higher than that of the AUC < 4 group. Decreased platelet and neutrophil counts of grade ≥3 occurred at higher rates in the AUC ≥ 4 group.

**Conclusion:**

The extrapolation of the Calvert formula has utility in calculating the CBDCA dosage for DeVIC ± R therapy, and therapeutic efficacy was increased by maintaining the AUC of CBDCA at ≥4.

## Introduction

Carboplatin (CBDCA), a type of platinum-containing drug, is widely used in the treatment of non-small cell lung cancer, ovarian cancer, cervical cancer and malignant lymphoma [1]. CBDCA is predominantly excreted by the kidneys, with approximately 70 % eliminated in the urine [2]. Accordingly, the Calvert formula

$${\text{dose }}\left( {\text{mg}} \right) = {\text{target area under the curve }}\left( {\text{AUC}} \right) \times \left( {{\text{glomerular filtration rate}} + 25} \right)$$

has demonstrated utility in calculating CBDCA dosage as it takes into account individual differences in renal function [3]. The utility of this method has also been documented in studies of Japanese patients [4]. CBDCA is rarely used in single-agent therapy. However, a combination therapy of CBDCA and paclitaxel (TC therapy) is used to treat ovarian cancer, and a combination therapy of CBDCA and pemetrexed is used to treat non-small cell lung cancer. In both cases, the Calvert formula is used to calculate CBDCA doses, with a target AUC of 5.0–7.5 [5] for TC therapy and 6 for CBDCA + PEM therapy [6] shown to be adequate.

DeVIC ± R therapy [7], ESHAP ± R therapy [8–10], EPOCH ± R therapy [11–13], ICE ± R therapy [14–17] and CHASE ± R therapy [18, 19] are often administered as salvage therapies for non-Hodgkin lymphoma, a type of haematological cancer, in Japan [20]. However, there is little evidence comparing the efficacies of each therapeutic regime. As a result, therapy is often chosen depending on the clinical condition of individual patients. DeVIC therapy, developed by Okamoto et al., combines dexamethasone (DEX), etoposide (ETP), ifosfamide (IFO) and CBDCA as a salvage therapy for recurrent or treatment-resistant non-Hodgkin lymphoma [7]. This therapy is unique in that it does not contain vincristine (VCR), doxorubicin (DXR) and cyclophosphamide (CPA), which are the components of CHOP therapy [21–23], generally considered the first-line therapy for non-Hodgkin lymphoma. DeVIC therapy is administered every 21 days for at least four cycles. The body surface area method has demonstrated utility in CBDCA dose calculation in DeVIC ± R therapy. Moskowitz et al. [15] reported response rate of 66.3 % when the target AUC of CBDCA was set at 5 in ICE therapy used for the treatment of non-Hodgkin lymphoma prior to peripheral blood stem cell transplantation and further demonstrated the utility of the Calvert formula in calculating the CBDCA dosage for haematological cancers. The present study included patients with non-Hodgkin lymphoma who had received DeVIC ± R therapy and was designed to retrospectively evaluate the AUC of CBDCA dosage (based on body surface area) administered using the Calvert formula and elucidate the relationship between AUC, therapeutic efficacy and adverse events. Accordingly, this was a preliminary study to determine the utility of the Calvert formula in calculating the CBDCA dosage for DeVIC therapy.

## Methods

### Patients

Patients included patients with relapsed or refractory non-Hodgkin lymphoma receiving DeVIC ± R as the initial therapy between January 2005 and March 2014 at the Department of Hematology, Fujita Health University Hospital. Patients with diseases other than non-Hodgkin lymphoma or those who did not receive more than one course of treatment were excluded. Patients with a creatinine clearance (CrCl) of >125 mL/min were excluded as CBDCA dosages may be overestimated by the Calvert formula in patients with abnormally low serum creatinine levels [24]. For DeVIC therapy, 40 mg/body of DEX, 1500 mg/m^2^ of IFO and 100 mg/m^2^ of ETP were each intravenously administered between days 1 and 3, and 300 mg/m^2^ of CBDCA was intravenously administered on day 1. Furthermore, 375 mg/m^2^ of rituximab was intravenously administered one day prior to the commencement of DeVIC therapy for the treatment of B-cell non-Hodgkin lymphoma. IFO, ETP and CBDCA dosages were reduced to 1000, 70 and 200 mg/m^2^, respectively, in patients aged 70 years or greater.

### Investigations

This was a retrospective study based on patient data collected from electronic patient files available in the databases of Fujita Health University Hospital. Surveyed parameters at treatment initiation included the following: age; gender; body surface area; histological images of lymphocytes; serum creatinine levels; platelet counts; haemoglobin (Hb) levels; neutrophil counts; dosages of CBDCA, IFO, ETP and DEX; and protocols for CBDCA dosing according to body surface area as indicated in the medical package insert of CBDCA. Surveyed items following the first cycle of DeVIC ± R therapy consisted of nadir of platelet counts, Hb levels, neutrophil counts and the occurrence and severity of thrombocytopaenia, anaemia and neutropaenia. CrCl was calculated using the Cockcroft and Gault method on the basis of peripheral blood serum creatinine levels. AUC values for CBDCA back-calculated from the actual administered dosage were used in the calculation formula shown below.

$${\text{AUC of CBDCA}} = {\text{Dose of carboplatin }}\left( {\text{mg}} \right)/\left( {{\text{CrCl}} + 25} \right)$$

The present study was conducted according to protocols approved by the Fujita Health University School of Medicine Epidemiological and Clinical Research Ethics Committee.

### Assessment

AUC was back-calculated using the Calvert formula on the basis of CBDCA dosages administered to patients receiving DeVIC ± R therapy. Patients were divided into two groups according to the calculated AUC, one group with AUC ≥ 4 and the other with AUC < 4, to compare treatment efficacy and safety. The International Working Group (IWG) Revised Response Criteria [25] were used to assess treatment efficacy after the final cycle. The National Cancer Institute—Common Terminology Criteria for Adverse Events version 4.0 was used to determine adverse events following the first cycle.

### Statistical analysis

Variables exhibiting a normal distribution were expressed as means ± standard deviations. Variables not exhibiting a normal distribution were given as medians with interquartile ranges. For comparison of values between the two groups, the unpaired *t* test was used for normally distributed variables, whereas the Mann–Whitney *U* test was used for non-normally distributed variables. The Chi-square test was used for ratio comparisons between the groups. For comparison of values between patients, the paired *t* test was used for normally distributed variables and the Wilcoxon signed-rank test was used for non-normally distributed variables. The Spearman’s rank correlation coefficient was used to study the correlation between AUC, actual dosage and therapeutic efficacy. Therapeutic efficacy was scored as follows: 4 points for complete response (CR), 3 points for partial response (PR), 2 points for stable disease (SD) and 1 point for progressive disease (PD). Univariate analysis was performed to identify factors influencing therapeutic efficacy in terms of CR or PR. Multivariate logistic regression analysis was performed for items with a risk rate of 10 % and below. The Hosmer–Lemeshow statistical test was used to validate the goodness of fit of the developed model. The Statistical Package for the Social Sciences (SPSS) version 22.0 (IBM Corporation, Armonk, NY, USA) was used for all statistical analyses. *P* values of <0.05 were considered statistically significant.

## Results

### Patient selection

We identified a total of 70 patients during the survey period. Of these, 13 patients were excluded: 6 with a CrCl of >125 mL/min, 3 with Hodgkin lymphoma, 1 with multiple myeloma, 1 who did not receive more than one treatment course and 2 with missing data. Accordingly, 57 patients were included in the final study sample.

### Validation of CBDCA dose calculation according to the AUC method

No significant difference was observed between the protocol dosage (median, 342 mg; range, 290–437 mg) and actual administered dosage (median, 336 mg; range, 300–420 mg; *P* = 0.309; Wilcoxon signed-rank test). In comparison, the AUC values back-calculated from the actual administered dosages (3.78 ± 0.95 min mg/mL) were significantly lower than the AUC values back-calculated from the protocol dosages (3.99 ± 0.73 min mg/mL; *P* = 0.038; paired *t* test). No significant differences in actual dosage or therapeutic efficacy were observed when the CR and PR groups (median, 247 mg/m^2^; range, 199–302 mg/m^2^) were compared with the PD and SD groups (median, 214 mg/m^2^; range, 194–299 mg/m^2^; *P* = 0.275; Wilcoxon signed-rank test). AUC values back-calculated from the actual administered dosage and therapeutic efficacy demonstrated that the AUC of the CR and PR groups (4.16 ± 0.88) was significantly higher than that of the PD and SD groups (3.53 ± 0.92; *P* = 0.013; unpaired *t* test). Furthermore, no correlation was observed between actual dosage and therapeutic efficacy when the two parameters were plotted with 4 as the maximum number of points for therapeutic efficacy (*ρ* = 0.165, *P* = 0.220; Spearman’s rank correlation coefficient). A weak correlation was observed between AUC and therapeutic efficacy (*ρ* = 0.302, *P* = 0.022; Spearman’s rank correlation coefficient).

### Group assignment

Table 1 shows the relationship between AUC values back-calculated from protocol dosages and response rate. A value of 4 was set as the AUC cut-off limit to divide the patients into two groups, according to response rates of more or <50 % to compare treatment efficacy. A total of 22 patients had an AUC ≥ 4 and 35 patients had an AUC < 4. Table 2 shows the baseline characteristics of the AUC ≥ 4 and AUC < 4 groups. Study parameters for which a significant difference between the groups was observed included body surface area, CrCl, CBDCA dosage, AUC back-calculated from the actual administered CBDCA dosage, actual CBDCA dosage divided by protocol CBDCA dosage and IFO and ETP dosages.

**Table 1:** Relationship between AUC and response rate

AUC of CBDCA dosage	Total	Effective (CR or PR)	Non-effective (SD or PD)	Response rate (%)
<2.5	4	0	4	0
2.5–<3.0	9	3	6	33.3
3.0–<3.5	11	2	9	18.2
3.5–<4.0	11	5	6	45.5
4.0–<4.5	8	4	4	50.0
4.5–<5.0	10	7	3	70.0
≥5.0	4	2	2	50.0

*AUC* area under the blood concentration–time curve, *CBDCA* carboplatin, *CR* complete response, *PR* partial response, *SD* stable disease, *PD* progressive disease

**Table 2:** Patient characteristics before DeVIC ± R therapy

	Total (*n* = 57)	AUC		*P* value
		<4 (*n* = 35)	≥4 (*n* = 22)	
Age	67.8 ± 11.3	67.6 ± 11.9	68.0 ± 10.5	0.88
Male gender (%)	57.9	60.0	54.5	0.68
Body surface area (m^2^)	1.44 ± 0.15	1.40 ± 0.14	1.49 ± 0.13	0.04
Histology (%)				
DLBCL	63.2	74.3	45.5	0.12
FL	8.8	5.7	13.6	
ENKL	5.3	5.7	4.5	
Others	24.6	14.3	40.9	
Creatinine clearance (mL/min)	67.4 ± 19.9	71.8 ± 21.1	60.4 ± 15.8	0.023
Course number of DeVIC	2.4 ± 1.3	2.2 ± 1.2	2.6 ± 1.4	0.18
CBDCA				
Dosage (mg)	336 (150–490)	300 (150–480)	412 (250–490)	0.0001
(mg/m^2^)	229 (109–328)	210 (109–310)	298 (191–328)	<0.0001
AUC (min·mg/mL)	3.78 ± 0.95	3.17 ± 0.55	4.76 ± 0.54	<0.0001
Actual dosage / protocol dosage	0.95 ± 0.19	0.88 ± 0.03	1.06 ± 0.02	0.0039
Dosage of IFO (mg/m^2^)	1225 ± 257	1159 ± 242	1330 ± 248	0.014
Dosage of ETP (mg/m^2^)	82.2 ± 18.0	76.5 ± 15.0	91.2 ± 18.0	0.0017
Dosage of dexamethasone (%)				
40 mg/body	77.2	71.4	86.4	0.19
<40 mg/body	22.8	28.6	13.6	
Rituximab combined therapy (%)	49.1	45.7	54.5	0.52
Dosage (mg)	374 (364–385)	373 (364–385)	374 (369–388)	0.54

*DLBCL* diffuse large B-cell lymphoma, *FL* follicular lymphoma, *ENKL* extranodal NK/T cell lymphoma, nasal type, *CBDCA* carboplatin, *AUC* area under the blood concentration–time curve, *IFO* ifosfamide, *ETP* etoposide

### Therapeutic efficacy

**Fig. 1:**
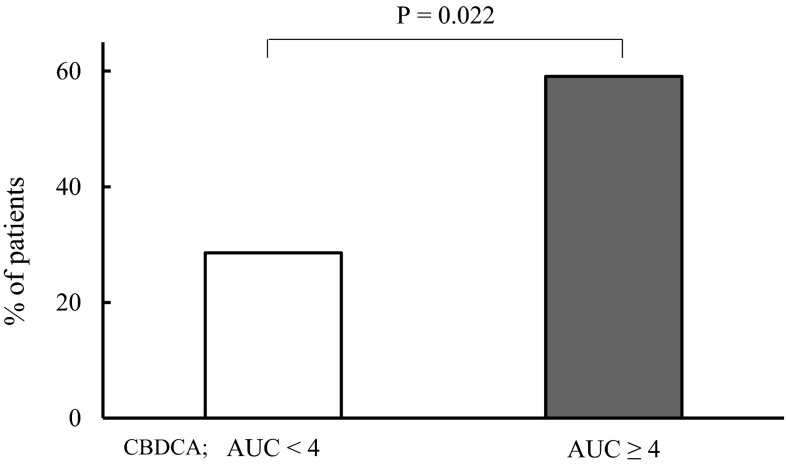
Percentage of patients with positive effect resulting in CR or PR for non-Hodgkin lymphoma after the final cycle of DeVIC ± R therapy. *CR* complete response, *PR* partial response, *CBDCA* carboplatin, *AUC* area under the blood concentration–time curve

The proportion of patients evaluated as having CR or PR in the AUC < 4 group was 28.6 %. In contrast, the corresponding proportion in the AUC ≥ 4 group was significantly higher (59.1 %; Fig. 1). Of these patients, the proportions receiving rituximab for B-cell non-Hodgkin lymphoma were 70.0 and 76.9 % for the AUC < 4 and AUC ≥ 4 groups, respectively (*P* = 0.71). The proportion of patients receiving rituximab evaluated as having CR or PR was significantly higher in the AUC ≥ 4 group (83.3 %) than in the AUC < 4 group (43.8 %; *P* = 0.034; *χ*^2^ test). Multivariate logistic regression analysis demonstrated a high AUC of CBDCA could become recognized as the factor independently associated with therapeutic efficacy in terms of CR or PR (Table 3). Rituximab administration and the number of DeVIC treatment courses were found to be strongly associated with therapeutic efficacy. On the other hand, age greater than or equal to 70 years, CBDCA dosage per body surface area and IFO and ETP dosage were not be associated with therapeutic efficacy.

**Table 3:** Factors influencing therapeutic efficacy resulting in CR or PR for non-Hodgkin lymphoma after the final cycle of DeVIC ± R therapy

	Univariate analysis		Multivariate analysis	
	Odds ratio (95 % CI)	*P* value	Odds ratio (95 % CI)	*P* value
Age ≥ 70 years	0.42	0.12		
	(0.14–1.26)			
Male gender	0.68	0.47		
	(0.23–1.97)			
Body surface area (m^2^)	29.14	0.094	0.01	0.41
	(0.56–>50)		(0.001–>50)	
DLBCL	2.24	0.17		
	(0.71–7.07)			
FL	0.34	0.35		
	(0.04–3.27)			
Creatinine clearance (mL/min)	0.99	0.60		
	(0.97–1.02)			
Dosage of CBDCA (mg/m^2^)	1.01	0.15		
	(1.00–1.02)			
AUC of CBDCA (min·mg/mL)	2.16	0.019	6.89	0.060
	(1.13–4.13)		(0.92–51.59)	
Actual dosage/protocol dosage of CBDCA	0.91	0.94		
	(0.06–13.64)			
Dosage of IFO (mg/m^2^)	1.00	0.44		
	(0.99–1.00)			
Dosage of ETP (mg/m^2^)	1.01	0.53		
	(0.98–1.04)			
Dosage of dexamethasone (mg/body)	1.09	0.042	1.06	0.32
	(1.00–1.19)		(0.95–1.19)	
Rituximab combined therapy	5.92	0.003	39.24	0.023
	(1.83–19.20)		(1.68–>50)	
Course number of DeVIC	12.00	<0.001	81.12	0.007
	(3.49–41.26)		(3.27–>50)	

Effective factors are analysed with multivariable logistic regression models

*CR* complete response, *PR* partial response, *DLBCL* diffuse large B-cell lymphoma, *FL* follicular lymphoma, *CBDCA* carboplatin, *AUC* area under the blood concentration–time curve, *IFO* ifosfamide, *ETP* etoposide

Hosmer–Lemeshow test, *P* = 0.88

### Haematotoxicity

To validate the haematotoxicity of DeVIC ± R therapy, baseline and nadir of platelet counts, Hb levels and neutrophil counts and frequency of haematotoxicity were investigated. Comparisons of data before and after treatment demonstrated that all values in both groups significantly declined (data not shown). Comparison of post-treatment haematological toxicities between the two groups demonstrated significantly lower neutrophil counts in the AUC ≥ 4 group than in the AUC < 4 group (Table 4). Analysis of the frequency of grade ≥3 haematotoxicity demonstrated a higher frequency of thrombocytopaenia and neutropaenia of grade ≥3 in the AUC ≥ 4 group than the AUC < 4 group (Table 5). The frequency of febrile neutropaenia was 22.7 % in the AUC ≥ 4 group and 25.7 % in the AUC < 4 group, with no statistically significant difference observed between the two groups (*P* = 0.80). Additionally, the frequency of grade 4 neutropaenia was 95.5 % in the AUC ≥ 4 group and 65.7 % in the AUC < 4 group, with a statistically significant difference observed between the two groups (*P* = 0.009). The frequency of grade 4 thrombocytopaenia was 45.5 % in the AUC ≥ 4 group and 37.1 % in the AUC < 4 group, with no statistically significant difference observed between the two groups (*P* = 0.53). No patient developed grade 4 anaemia during the present study. Multivariate logistic regression analysis did not demonstrate an association between AUC of CBDCA and grade 4 haematological toxicity (data not shown).

**Table 4:** Changes in peripheral blood cell count before and after the first cycle of DeVIC ± R therapy

	Total (*n* = 57)	AUC		*P* value
		<4 (*n* = 35)	≥4 (*n* = 22)	
Platelet (×10^4^/μL)				
Baseline	16.8 (1.4–47.0)	18.1 (1.4–47.0)	14.3 (4.5–42.2)	0.14
Nadir	3.0 (0.6–26.1)	4.2 (0.7–26.1)	2.7 (0.6–13.9)	0.13
Haemoglobin (μg/dL)				
Baseline	10.4 ± 2.1	10.0 ± 1.9	11.1 ± 4.9	0.045
Nadir	8.4 ± 2.0	8.2 ± 3.6	8.5 ± 4.9	0.57
Neutrophil (/μL)				
Baseline	3290 (884–16,356)	2716 (990–15,614)	3682 (884–16,356)	0.35
Nadir	108 (5–6384)	310 (5–6384)	47 (5–868)	0.001

*AUC* area under the blood concentration–time curve

**Table 5:** Incidence of adverse events related to peripheral blood cell count before and after the first cycle of DeVIC ± R therapy

Grade	Total (*n* = 57)	AUC		*P* value
		<4 (*n* = 35)	≥4 (*n* = 22)	
Thrombocytopaenia (%)				
<3	35.1	45.7	18.2	0.034
≥3	64.9	54.3	81.8	
Anaemia (%)				
<3	49.1	45.7	54.5	0.52
≥3	50.9	54.3	45.5	
Neutropaenia (%)				
<3	10.5	17.1	0	0.040
≥3	89.5	82.9	100.0	

*AUC* area under the blood concentration–time curve

## Discussion

In Japan, medical package inserts for CBDCA recommend a dose calculation method according to the body surface area. However, recent studies [5, 6, 15] have reported the utility of extrapolating the Calvert formula in calculating the dosage of CBDCA. Therefore, we performed the present preliminary study to evaluate whether the Calvert formula can be extrapolated for CBDCA dose calculation in DeVIC ± R therapy. As a result, we demonstrate the greater utility of calculating CBDCA dosage according to AUC instead of body surface area. A pilot study of DeVIC therapy reported that the CR rate is 24 % for a CBDCA dosage of 300 mg/m^2^ (reduced for patients aged 70 years or older) [7], with a mean CR rate among all study individuals of 26.3 %, comparable to that observed in the present study. However, the CR rate for the AUC ≥ 4 group was substantially higher (36.0 %). We were able to validate the increase in therapeutic efficacy by setting the AUC of CBDCA at ≥4, with a significantly higher response rate (59.1 %) obtained compared to that in the AUC < 4 group. Furthermore, rituximab was found to have a synergistic effect.

Patient profiles at baseline were characterized by several factors that may influence the results of comparisons between the AUC < 4 and AUC ≥ 4 groups. Therefore, multivariate logistic regression analysis was performed to identify factors associated with therapeutic efficacy, which thus influence CR and PR. Following this analysis, a high AUC of CBDCA could become recognized as the factor independently associated with therapeutic efficacy. Creatinine clearance may not have been identified as a factor associated with therapeutic efficacy on multivariate logistic regression analysis because the concentration of CBDCA dosage determined from the body surface area regardless of creatinine clearance was sufficient to render therapeutic effects. On the other hand, rituximab administration and the number of DeVIC treatment courses were found to be strongly associated with therapeutic efficacy. However, these results demonstrate the AUC of CBDCA, calculated by the extrapolation of the Calvert formula, was taken into consideration although the influence of other factors (such as rituximab administration and the number of DeVIC treatment courses) was strong. Although the results of the present study demonstrate the validity of CBDCA dose calculation according to the AUC method, the low number of patients included in the multivariate logistic regression analysis may have reduced the statistical power. As mentioned in the introduction, there is little evidence regarding the efficacy of individual salvage therapies, and it is conventional practice for therapies to be chosen at the physician’s discretion depending on the patient’s clinical condition; therefore, it is difficult to make a direct comparison of effectiveness. Nevertheless, the CR rate when calculating CBDCA dosage per body surface area for DeVIC ± R therapy (26.3 %) was inferior to that for DHAP therapy (31 %) developed around the same time [26] and ESHAP therapy (37 %) [8]. In contrast, the CR rate could be improved to 36 % in the present study by setting the AUC at ≥4 and extrapolating the Calvert formula for CBDCA dosage calculation. Furthermore, we demonstrated the validity of CBDCA dose calculation according to the AUC method. Jodrell et al. [27] studied single-agent CBDCA therapy in 1028 ovarian cancer patients and demonstrated a positive correlation between response rates and an AUC of up to 5; however, the increase in response rate diminished after AUC values became >5. The sample size in the present study was small; therefore, we were unable to identify a specific upper limit of the AUC. The use of ESHAP therapy remains controversial as the addition of rituximab has no effect on overall response rate, CR rate or actuarial curves at 5 years [10]. On the other hand, the CR rate for ICE therapy without rituximab has been reported as 27 %; however, a significantly higher CR rate (53 %) was observed with the addition of rituximab [16]. Accordingly, rituximab appears to have varying effects depending on the treatment method. As demonstrated by the results of the present study, response rates may be improved by the addition of rituximab when choosing DeVIC therapy for B-cell malignant lymphoma.

The frequency of thrombocytopaenia and leukopaenia has been reported to increase when the AUC of CBDCA increases [3, 16, 28]. In the present study, the rate of grade ≥3 thrombocytopaenia and neutropaenia was high in the AUC ≥ 4 group and particularly prominent for neutropaenia, which was grade ≥3 in all patients. In general, the frequency of grade ≥3 haemopaenia is high following chemotherapy for haematological cancer. Oki et al. [18] reported grade ≥3 neutropaenia and thrombocytopaenia in all cases in a study on CHASER therapy in 38 patients. In the present study, no difference was observed between the AUC ≥ 4 group (22.7 %) and the AUC < 4 group (25.7 %) in terms of the development of febrile neutrophilia, with both rates in the 20 % range. Avilés et al. [10] reported the frequencies of grade ≥3 neutropaenia after ESHAP and ESHAP + R therapies as 30 and 32 %, respectively, whereas a study conducted by Martin et al. using the same treatment methods [9] reported rates of febrile neutropaenia as 33.8 and 33.3 %, respectively. The rate of febrile neutropaenia in the present study was lower than that in the study reported by Martin et al. on ESHAP ± R therapy. Oki et al. [18] reported a very high rate of febrile neutropaenia (78 %) in a study of CHASER therapy. These numbers cannot be directly compared as patients and drugs included differed between studies; however, it is unlikely that an AUC cut-off value for CBDCA of ≥4 for DeVIC therapy increases the risk of severe haematopaenia compared with other treatment methods. Furthermore, we demonstrated that AUC of CBDCA did not influence a risk factor for grade 4 haematological toxicity. These findings suggest that setting an AUC cut-off value of ≥4 increases the risk of myelosuppression; however, this can be satisfactorily controlled clinically. However, Kewalramani et al. [16] reported grade 3 or 4 haematologic toxicity as the primary reason for delay when the target AUC of CBDCA was set at 5 for RICE therapy administered before autologous stem cell transplantation for DLBCL. A previous detailed study of four patients with an AUC of 5 or above reported a frequency of febrile neutropaenia of 50 %. Although this finding is of limited value due to the small sample size, it has been postulated that an AUC substantially greater than 4 reflects an increase in the risk of severe adverse events.

There were many limitations to the present study. First, the small sample size of 57 reflects the low proportion of patients with poor responses to first-line treatment and the variety of treatment options for patients in such scenarios. This issue was avoided by the use of less strict exclusion criteria and multivariate analysis to increase statistical precision. Second, it was difficult to evaluate differences in non-haematological toxicity between the two groups due to the retrospective design of the present study. Third, dosages of administered anti-cancer drugs other than CBDCA differed between patients, although we believe that this difference was controlled for using multivariate logistic regression analysis. Finally, therapeutic efficacy was evaluated upon the completion of DeVIC ± R therapy, while the evaluation of changes in blood cell count was limited to the first round of treatment as administration rounds differed between patients.

The results of the present study indicate that the response rate of DeVIC ± R therapy in non-Hodgkin lymphoma depends on the AUC of CBDCA. We further clarify that the response rate increases at an AUC of ≥4. Furthermore, we suggested that an AUC of ≥4 increases the risk of myelosuppression; however, this can be satisfactorily controlled clinically. A more efficacious target AUC value has been demonstrated to be 5 in ICE ± R therapy, a separate salvage therapy for non-Hodgkin lymphoma [16, 17]. On the basis of these observations, we posit an AUC of ≥4 as an adequate target range for AUC in DeVIC ± R therapy. However, as this was a retrospective study, we are unable to propose a specific target AUC. Further prospective studies using uniform conditions are required to accurately identify target AUCs.
